# Stem Cells Therapy for COVID-19: A Systematic Review and Meta-Analysis

**DOI:** 10.3389/fmed.2021.737590

**Published:** 2021-11-29

**Authors:** Erfan Arabpour, Sina Khoshdel, Negin Tabatabaie, Ali Akhgarzad, Moein Zangiabadian, Mohammad Javad Nasiri

**Affiliations:** ^1^Student Research Committee, School of Medicine, Shahid Beheshti University of Medical Sciences, Tehran, Iran; ^2^Department of Microbiology, School of Medicine, Shahid Beheshti University of Medical Sciences, Tehran, Iran

**Keywords:** stem cell, mesenchymal stem cell, cell therapy, COVID-19, SARS-CoV-2, 2019 novel coronavirus

## Abstract

**Introduction:** Vaccination seems to be a good solution for preventing and controlling coronavirus disease (COVID-19) pandemic, but still there are some challenges in COVID-19 vaccination. Investigating new therapeutic options for COVID-19 is necessary. The current study aimed to evaluate the safety and efficacy of stem cells in treating patients with COVID-19.

**Methods:** We reviewed the relevant scientific literature published up to April 1, 2021. The pooled risk ratio (RR) with 95% CI was assessed using a fixed or random-effect model. We considered *P* < 0.05 as statistically significant for publication bias. Data were analyzed by Comprehensive Meta-Analysis software, Version 2.0 (Biostat, Englewood, NJ).

**Results:** After reviewing 1,262 records, we identified 10 studies that met the inclusion criteria. The analysis showed that stem cell therapy could significantly reduce the mortality rate (RR 0.471, 95% CI: 0.270–0.821) and morbidity (RR 0.788, 95% CI: 0.626–0.992) in patients with COVID-19; compared with the control group.

**Conclusions:** The present study suggests that stem cell therapy has a remarkable effect on reducing mortality and morbidity of patients with COVID-19. Further large-scale studies are needed to approve these results. Defining a protocol for stem cell therapy in patients with COVID-19 can lead to achieving the best clinical outcomes.

## Introduction

Since the primary detection of coronavirus disease (COVID-19) in December 2019 in Wuhan, China, it has caused more than 175 million infected cases and 3.7 million deaths up to June 10, 2021, all over the world (1). COVID-19 pandemic has been putting a massive mental, health, social, and economic burden on individuals and societies (2, 3).

Vaccination has been shown to be a good solution for this problem. However, vaccines have some limitations, including, they are for prevention and not for treatment. Their protection is not 100%, and their distribution worldwide is unfair (4, 5).

A broad spectrum of treatments was introduced for COVID-19, such as remdesivir, favipiravir, corticosteroids, tocilizumab, hydroxychloroquine, and convalescent plasma therapy (4, 5). Furthermore, another therapeutic option is stem cell therapy (6, 7). Stem cells are used in the treatment of a wide variety of diseases, from autoimmune diseases (8) to heredity (9) and infectious diseases (10). Mechanisms, which include immunomodulation, regenerative ability, clearance of alveolar fluid, and preventing thrombotic events, drive the researchers to use these cells to treat COVID-19 (11). Mesenchymal stem cells (MSCs) are shown to be effective in reducing inflammation by releasing chemokines (CCL5, CXCL9,10,11) and other factors [transforming growth factor-beta (TGF-β), nitric oxide (NO)/indoleamine 2,3-dioxygenase (IDO), prostaglandin E2 (PGE2)] in their secretomes. The immunomodulation mechanisms include: (a) inhibiting B cells, T cells, and natural killer (NK) cells proliferation and activity; (b) inhibiting maturation and antigen-presenting of dendritic cells; (c) enhancing M2 macrophage activation; and (d) restraining cytokine storm (12–14). This anti-inflammatory property has such importance that MSCs are used to prevent graft vs. host disease (GvHD) in many organ transplantations (15). Some studies reported that stem cells could reduce mortality rate and improve pulmonary function and disease remission in patients with COVID-19. However, a comprehensive analysis on this issue has not yet been performed. The current study aimed to evaluate the safety and efficacy of stem cells in treating patients with COVID-19.

## Methods

This study was conducted and reported according to the PRISMA statement (16).

### Search Strategy

We searched Pubmed/Medline, Embase, Scopus, Clinicaltrials.gov, and gray literature (i.e., google scholar and L·OVE) for studies reporting the efficacy/effectiveness of stem cells in patients with COVID-19, published up to April 1, 2021. The search terms were the following: stem cell, progenitor cell, mesenchymal stem cell, cell therapy, COVID-19, SARS-CoV-2. Only studies written in English were selected.

### Study Selection

The records found through database searching were merged, and the duplicates were removed using EndNote X7 (Thomson Reuters, New York, NY, USA). Two reviewers independently screened the records by title/abstract and full texts to exclude those unrelated to the study topic. The studies included met the following inclusion criteria: (i) patients were diagnosed with COVID-19 based on the WHO criteria; (ii) patients were treated with stem cells; and (iii) treatment outcomes were recorded. Conference abstracts, editorials, reviews, and experimental studies on animal models were excluded.

### Data Extraction

Two reviewers designed a data extraction form. These reviewers extracted data from all eligible studies, and differences were resolved by consensus. The following data were extracted: first author name; year of publication; type of epidemiological study, country/ies where the research was conducted; treatment protocols, demographics, adverse effects, and outcomes.

### Quality Assessment

The checklists provided by the Joanna Briggs Institute (JBI) for randomized controlled trials (RCTs) were used to perform the quality assessment (17).

### Statistical Analysis

The pooled risk ratios (RRs) with 95% CI were assessed using random or fixed-effect models. The random-effects model was used because of the estimated heterogeneity of the true effect sizes. The between-study heterogeneity was assessed by Cochran's Q and the *I*^2^ statistic. Publication bias was evaluated statistically by using Egger's and Begg's tests as well as the funnel plot (*p* < 0.05 was considered indicative of statistically significant publication bias; funnel plot asymmetry also suggests bias). All analyses were performed using “Comprehensive Meta-Analysis” software, Version 2.0 (Biostat, Englewood, NJ).

## Results

**Figure 1:**
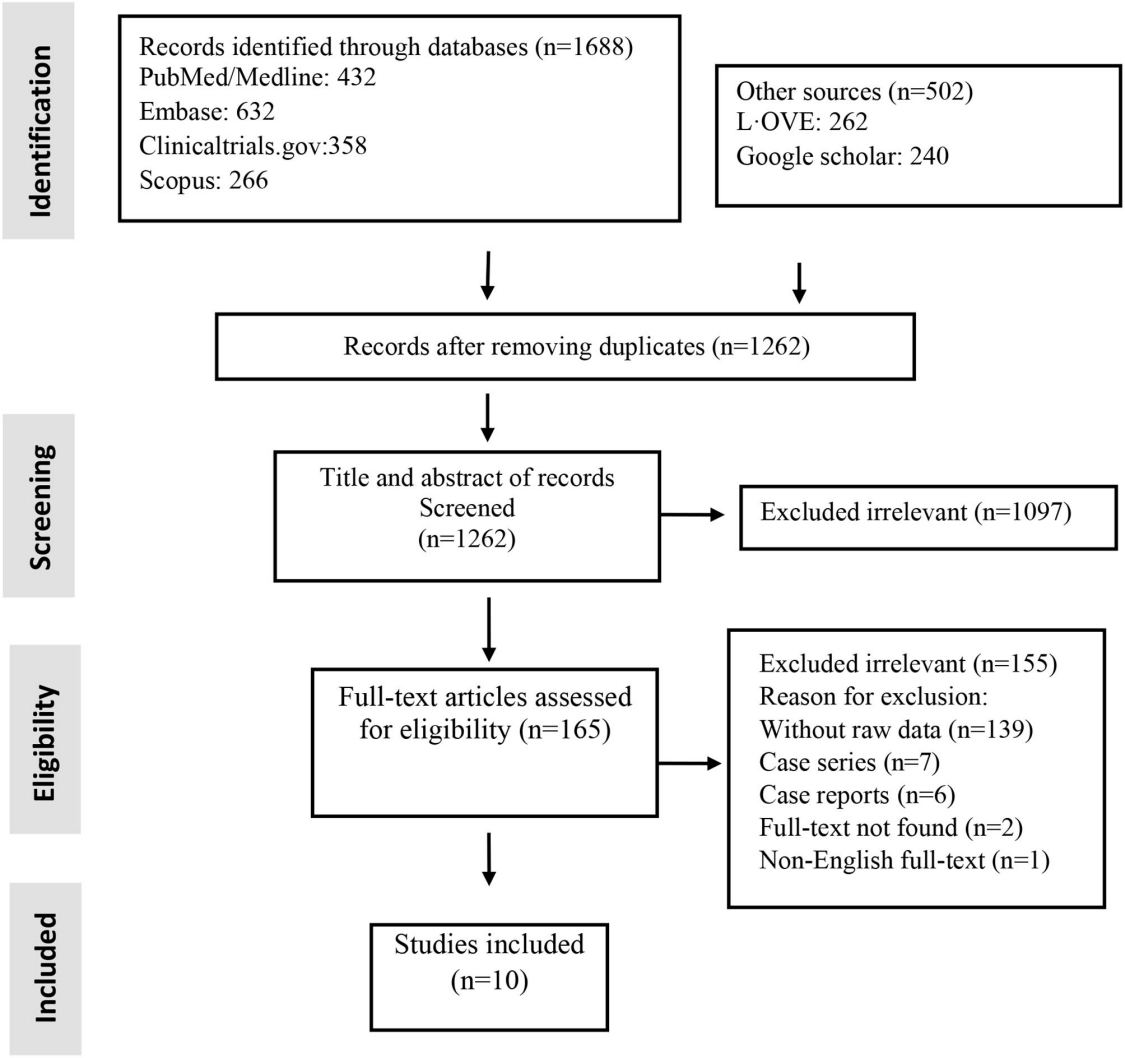
Flow chart of study selection for inclusion in the systematic review and meta-analysis.

Studies included and excluded through the review process are summarized in Figure 1. A total of 1,262 records were found in the initial search; after removing duplicate articles, and full-text review, 10 were chosen (Figure 1). Of the included studies, there were five non-randomized clinical trials and five RCTs (Table 1). Totally, 184 patients underwent stem cell therapy, while 161 patients took part as controls. Most studies assessed mortality, morbidity, adverse event (AE) and severe adverse event (SAE), pulmonary function, imaging changes, systematic symptoms, and inflammatory markers.

**Table 1:** Characteristics of included studies.

**References**	**Study design**	**Country**	**Case definition**	**Control definition**	**Morbidity definition**
Meng et al. (18)	Non-randomized clinical trial	China	Received UC-MSC and standard COVID-treatment regimens	Received standard COVID-treatment regimens	Mechanical ventilation
Häberle et al. (19)	Non-randomized clinical trial	Germany	Received MSC transplantation	Received standard therapy	Acute kidney injury (any level)
Leng et al. (20)	Non-randomized clinical trial	China	Received MSCs transplant	Received placebo control	Not discharging from hospital until the end of the study
Nesrin et al. (21)	Non-randomized clinical trial	Turkey	Received MSCs transplantation	Reviewed without MSCs transplantation	Not discharging from ICU until the end of the study
Xu et al. (22)	Non-randomized clinical trial	China	Received MSC infusion and concomitant medication	Received concomitant medication	Multiple organ dysfunction syndrome (MODS)
Giacomo et al. (23)	RCT	USA	Received UC-MSC treatment	Received control treatment	Any serious adverse event
Lei et al. (24)	RCT	China	Received UC-MSC treatment	Received control treatment	Any adverse event
Shu et al. (25)	RCT	China	Received UC-MSC treatment	Received placebo treatment	No clinical improvement after 28 day
Gina Marcela Torres et al. (26)	RCT	UAE	Received COVID 19 standard care plus nebulization with NHPBSC	Received COVID 19 standard care	Continuous renal replacement therapy for AKI with hemodialysis
Dynasty (27)	RCT	Russia	Received standard therapy and MSCs derived exosomes	Received standard therapy and inhalation placebo solution	Serious and Non-serious Adverse Events During Trial

*AKI, acute kidney injury; ICU, intensive care unit; MSC, mesenchymal stem cell; NHPBSC, non-hematopoietic peripheral blood stem cell; RCT, randomized controlled trial; UC-MSC, umbilical cord-derived MSC*.

### Quality of Included Studies

Based on the JBI checklist for experimental studies, the included studies had a low risk of bias (Table 2).

**Table 2:** Quality assessment of experimental studies.

**References**	**Was true randomization used for assignment of participants to treatment groups?**	**Was allocation to treatment groups concealed?**	**Were treatment groups similar at the baseline?**	**Were participants blind to treatment assignment?**	**Were those delivering treatment blind to treatment assignment?**	**Were outcomes assessors blind to treatment assignment?**	**Were treatment groups treated identically other than the intervention of interest?**	**Was follow up complete and if not, were differences between groups in terms of their follow up adequately described and analyzed?**	**Were participants analyzed in the groups to which they were randomized?**	**Were outcomes measured in the same way for treatment groups?**	**Were outcomes measured in a reliable way?**	**Was appropriate statistical analysis used?**	**Was the trial design appropriate, and any deviations from the standard RCT design accounted for in the conduct and analysis of the trial?**
Meng et al. (18)	No	No	Yes	No	No	No	Yes	Yes	Yes	Yes	Yes	Yes	No
Häberle et al. (19)	No	No	Yes	No	No	No	Yes	Yes	Yes	Yes	Yes	Yes	No
Leng et al. (20)	No	Yes	Yes	No	No	No	Yes	Yes	Yes	Yes	Yes	Yes	No
Nesrin et al. (21)	No	No	Yes	No	No	No	Yes	Yes	Yes	Yes	Yes	Yes	No
Xu et al. (22)	No	No	Yes	No	No	No	Yes	Yes	Yes	Yes	Yes	Yes	No
Giacomo et al. (23)	Yes	Yes	Yes	Yes	Yes	Yes	Yes	Yes	Yes	Yes	Yes	Yes	Yes
Lei et al. (24)	Yes	Yes	Yes	Yes	Yes	Yes	Yes	Yes	Yes	Yes	Yes	Yes	Yes
Shu et al. (25)	Yes	Yes	Yes	No	No	No	Yes	Yes	Yes	Yes	Yes	Yes	Yes
Gina Marcela Torres et al. (26)	Yes	Yes	Yes	Na	Na	Na	Yes	Yes	Yes	Yes	Yes	Yes	Yes
Dynasty (27)	Yes	Yes	Yes	Yes	Yes	Yes	Yes	Yes	Yes	Yes	Yes	Yes	Yes

*NA, Not applicable*.

### Patient Characteristics

The origins of studies were six countries: China (*n* = 5) (18, 20, 22, 24, 25), United States (*n* = 1) (23), Germany (*n* = 1) (19), Turkey (*n* = 1) (21), UAE (*n* = 1) (26), and Russia (*n* = 1) (27). In these 10 studies, the age range for stem cell groups was 39–64 and 48–64 for control groups. Except for two studies (22, 27), which did not report comorbidities of the study population, diabetes mellitus (34 of 138 cases) and hypertension (40 of 138 cases) were the most common reported comorbidities (Table 3).

**Table 3:** Patients' characteristics.

**References**	**Total,** ***n*** **(sc; ctrl)**	**Mean age (sc; ctrl)**	**Comorbidities** **(sc; ctrl)**	**Follow up time**	**Covid-19 severity (sc; ctrl)**	**Covid-19 detection method**
Meng et al. (18)	18 (9; 9)	45; 50	(HTN: 2; 1), (Diabetes: 1; 0), (Fatty liver disease 1; 0), (Asthma: 0; 1)	28 days	(5 moderate, 4 severe; 5 moderate, 4 severe)	RT-PCR
Häberle et al. (19)	23 (5; 18)	39; 59	(Arterial hypertension: 1: 13), (CHF: 0; 2), (Coronary heart disease: 0; 2), (Chronic atrial fibrillation: 0; 2), (Pulmonary diseases: 0; 1), (Diabetes: 0; 2), (Smoker: 0; 3)	MSC group: 49 days (IQR 18–54); Control group: 15 days (IQR 6–29)	(5 severe; 18 severe)	NM
Leng et al. (20)	10 (7; 3)	57; 65	(HTN: 1; NM)	14 days	(4 severe, 2 common, 1 critically ill; 3 severe)	RT-PCR
Nesrin et al. (21)	11 (8; 3)	64; 68	(HTN: 4; 1), (Diabetes: 4, 1)	7–41 days	(6 critical severe, 2 severe; 3 critical severe)	RT-PCR and thorax CT
Xu et al. (22)	44 (26; 18)	58; 61	NM	1 month	(16 severe, 10 critical; 10 severe, 8 critical)	PCR
Giacomo et al. (23)	24 (12; 12)	58; 58	(Diabetes: 5; 6), (HTN: 7; 9), (Obesity: 11; 5), (cancer: 0; 1), (Heart disease: 1; 3)	31 days	(3 mild to moderate, 9 moderate to severe; 3 mild to moderate, 9 moderate to severe)	RT-PCR
Lei et al. (24)	100 (65; 35)	60; 59	(HTN: 17; 10), (Diabetes: 12; 5), (Chronic bronchitis: 2; 3), (COPD: 2; 0)	28 days	(65 severe; 35 severe)	RT-PCR
Shu et al. (25)	41 (12; 29)	61; 57	(Diabetes 3; 5), (HTN: 3; 6)	28 days	(12 severe; 29 severe)	NM
Gina Marcela Torres et al. (26)	44 (20; 24)	49; 48	(Smoker: 1; 0), (Diabetes: 9; 7), (HTN: 5; 6), (Dislipidemia: 3; 1), (Cardiac disease: 1; 1), (Respiratory diseases: 2; 1)	SC group: 13–45 days; Control group: 11–126 days	(20 critical; 24 critical)	NM
Dynasty (27)	30 (20; 10)	50; 53	NM	70 days	NM	PCR or antibody test

*CHF, congestive heart failure; COPD, chronic obstructive pulmonary disease; CT, computed tomography; CTRL, control; HTN, hypertension; MSC, mesenchymal stem cell; NM, not mentioned; PCR, polymerase chain reaction; RT-PCR, real-time polymerase chain reaction; SC, stem cell*.

### Intervention Characteristics

Among the 10 studies, four used umbilical cord-derived MSC (UC-MSC) (18, 23–25), one pericytes-derived MSC (21), one menstrual blood-derived MSC (22), one non-hematopoietic peripheral blood stem cells (NHPBSC) (26), and one used MSCs-derived exosomes (27). Two studies did not report the type of MSC (19, 20). In dosing, four studies administered 1–2 × 10^6^ cells per kg of body weight (19–21, 25), four administered 30–120 million cells per infusion (18, 22–24), one used 0/5–2 × 10^10^ of MSC-derived exosomes per administration (27), and one did not report stem cell dose (26). Of the 184 patients in the stem cell group, 27 received a single dose, 35 received two doses, 102 received three doses of therapy, and 20 received therapy for 20 doses. Route of delivery was intravenous (IV) in nine studies and inhalation in one study (27) (Table 4).

**Table 4:** Intervention characteristics.

**References**	**Stem cell source**	**Stem cell dose (cells per kg)**	**Frequency**	**Route of delivery**
Meng et al. (18)	UC-MSC	Total dose: 3 × 10^7^	3	Intravenous infusion
Häberle et al. (19)	NM	1 × 10^6^	2 for three patients, 3 for two patients	Intravenous infusion
Leng et al. (20)	NM	1 × 10^6^	1	Intravenous infusion
Nesrin et al. (21)	Pericytes derived MSC	1 × 10^6^	1	Intravenous infusion
Xu et al. (22)	Menstrual blood-derived MSCs	Total dose: 3 × 10^7^	3	Intravenous infusion
Giacomo et al. (23)	UC-MSC	Total dose: 100 ± 20 × 10^6^ in 50 ml	2	Intravenous infusion
Lei et al. (24)	UC-MSC	Total dose: 4 × 10^7^	3	Intravenous infusion
Shu et al. (25)	UC-MSC	2 × 10^6^	1	Intravenous infusion
Gina Marcela Torres et al. (26)	Non-hematopoietic peripheral blood stem cells	NM (just reported 10 ml solution)	2	Intravenous infusion
Dynasty (27)	MSCs derived exosomes	Total dose: 3 ml special solution contained 0.5–2 × 10^10^ exosomes	20 (twice a day for 10 days)	Inhalation

*KG, kilogram; ml, milliliter; MSC, mesenchymal stem cell; NM, not mentioned; UC-MSC, umbilical cord-derived MSC*.

### Primary Outcomes: Safety

#### Severe Adverse Events

None of the 10 studies reported treatment-related SAEs. Furthermore, none of the 13 deaths in stem cell groups were related to cell infusion (Table 5).

**Table 5:** Primary outcome: safety.

**References**	**Treatment-related adverse events**	**Treatment-related serious adverse events**
Meng et al. (18)	Two patients in the MSC group developed fever and transient facial flushing immediately on infusion, which resolved spontaneously within 4 h. Another patient with the moderate disease had a transient fever (38°C) within 2 h that resolved within 24 h	None
Häberle et al. (19)	None	None
Leng et al. (20)	No acute infusion-related, allergic reaction, delayed hypersensitivity, or secondary infection was detected	None
Nesrin et al. (21)	No adverse effects were observed related to infusion or allergic reactions, secondary infection, or life-threatening adverse events in MSC patients	None
Xu et al. (22)	54 AE occurred in 20 of 26 MSC group, and 56 AE occurred in 18 of 18 control group during the whole trial	None
Giacomo et al. (23)	No definite or probable TR-AE in both groups. The only reported adverse event in the MSC group occurred in a patient with bradycardia, who experienced aggravating of bradycardia and needed transient vasopressor treatment	None
Lei et al. (24)	No MSC-related predefined respiratory or hemodynamic adverse events were observed. The incidence of adverse events during the whole trial was similar between the MSC group (55.38%) and the control group (60%)	None
Shu et al. (25)	All MSC group patients had no adverse reactions (such as rash, allergic reaction, and febrile reaction after infusion)	None
Gina Marcela Torres et al. (26)	None	None
Dynasty (27)	None	None

*AE, adverse event; h, hour; MSC, mesenchymal stem cell; SAE, severe adverse event; TR-AE, treatment-related adverse event*.

#### Adverse Events

Two studies reported minimal infusion-related AE in the stem cell group. Meng et al. (18) reported two cases of transient facial flushing and fever immediately on infusion and one case of transient fever within 2 h; all of these AEs resolved without intervention. Giacomo et al. (23) reported only one AE in the MSC group, who needed an increase in vasopressor dose because of exacerbating of bradycardia. Lei et al. and Xu et al. (22, 24) had assessed AEs during the trial for COVID-19 (not for infusion). In both the studies, the incidence of AE in the MSC group was less than in the control group. All other seven studies reported no cell infusion-related AE (Table 5).

### Secondary Outcome: Efficacy

#### Mortality

**Figure 2:**
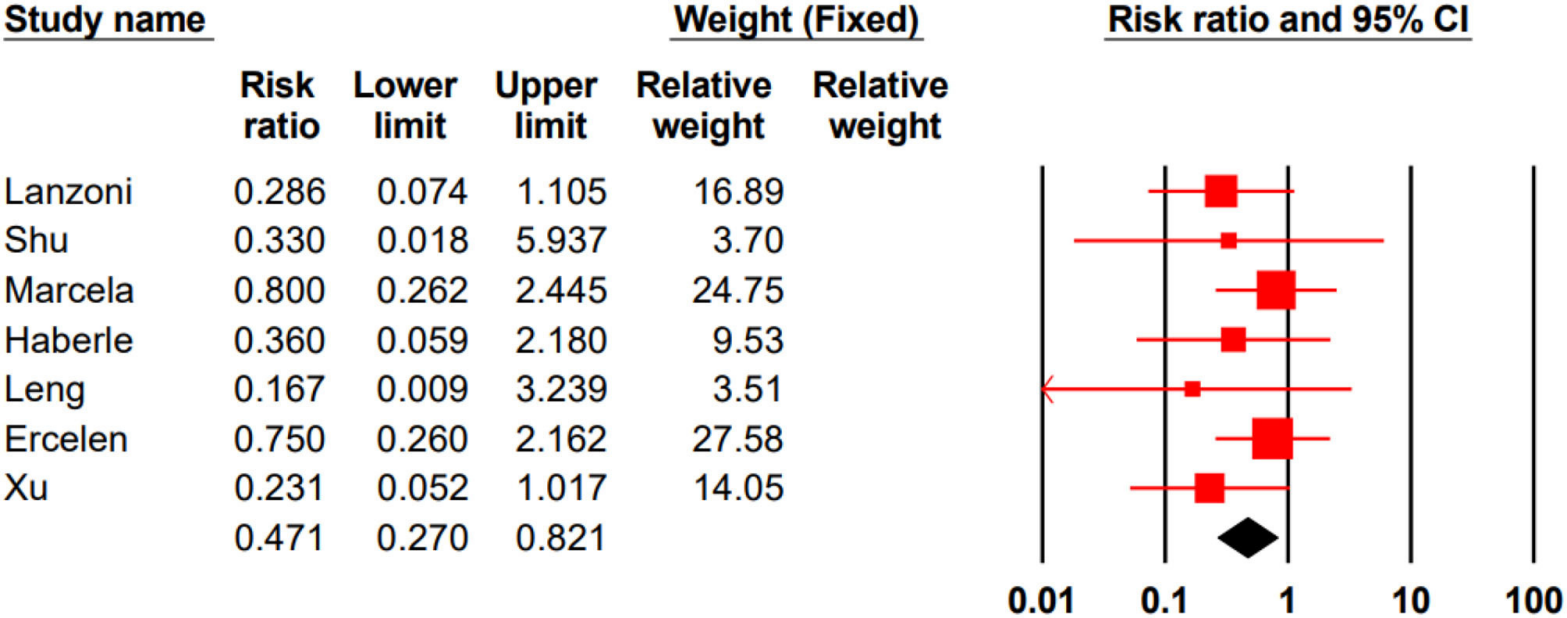
Pooled RR of mortality analysis.

In three studies, all participated patients survived (18, 24, 27). In seven remained studies, the total mortality rate for the stem cell group was %14/4 (13 of 90) and %32/7 (35 of 107) for the control group. Our meta-analysis showed that stem cell therapy could significantly decrease the mortality rate in patients with COVID-19 (RR 0.471, 95% CI 0.270–0.821) (Figure 2).

**Figure 3:**
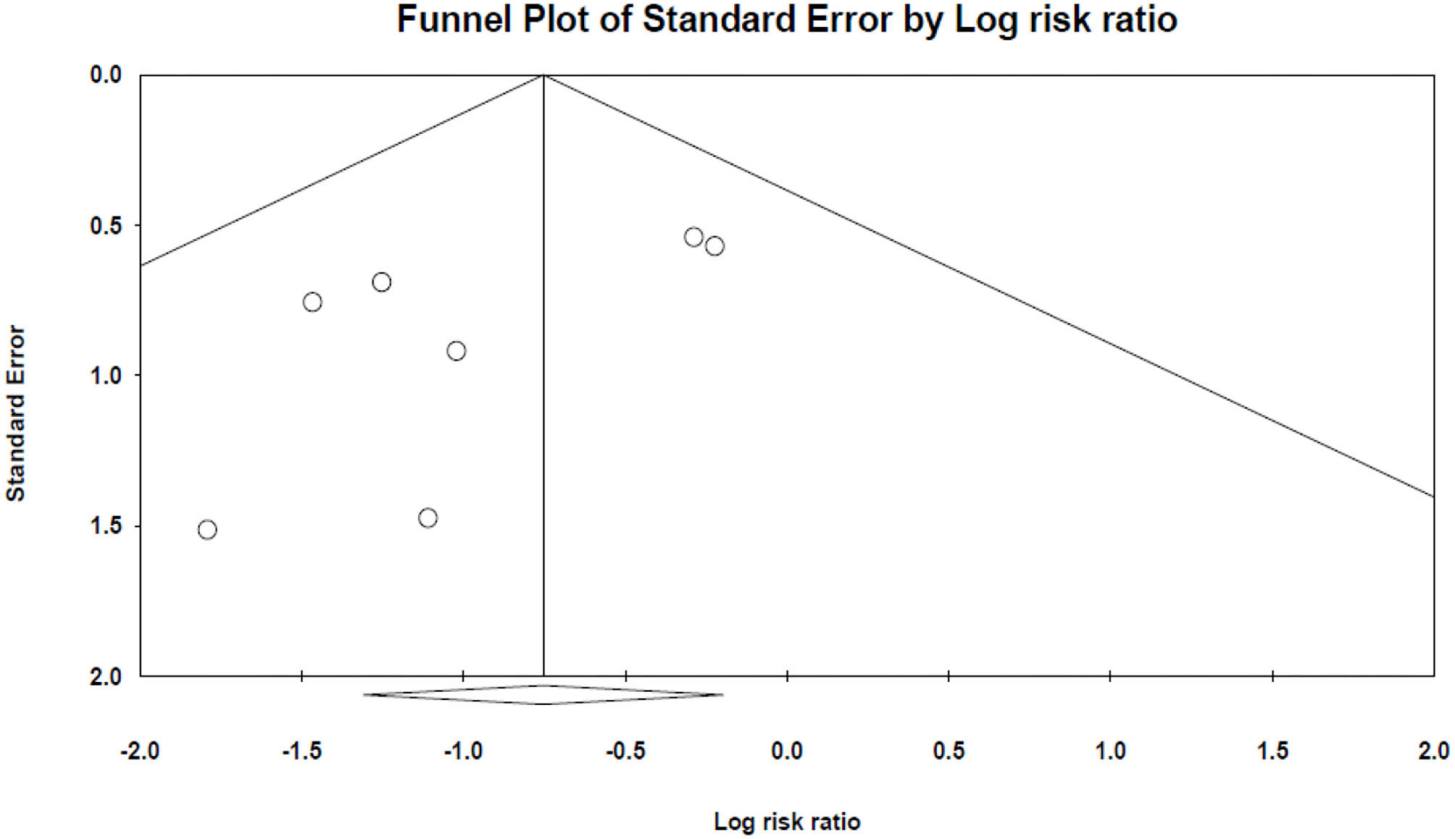
The funnel plot of mortality analysis.

The results of Egger's and Begg's tests did not show any evidence of publication bias (The *P*-value of Egger's tests was 0.1, and Begg's tests was 0.5) (Figure 3).

#### Morbidity

**Figure 4:**
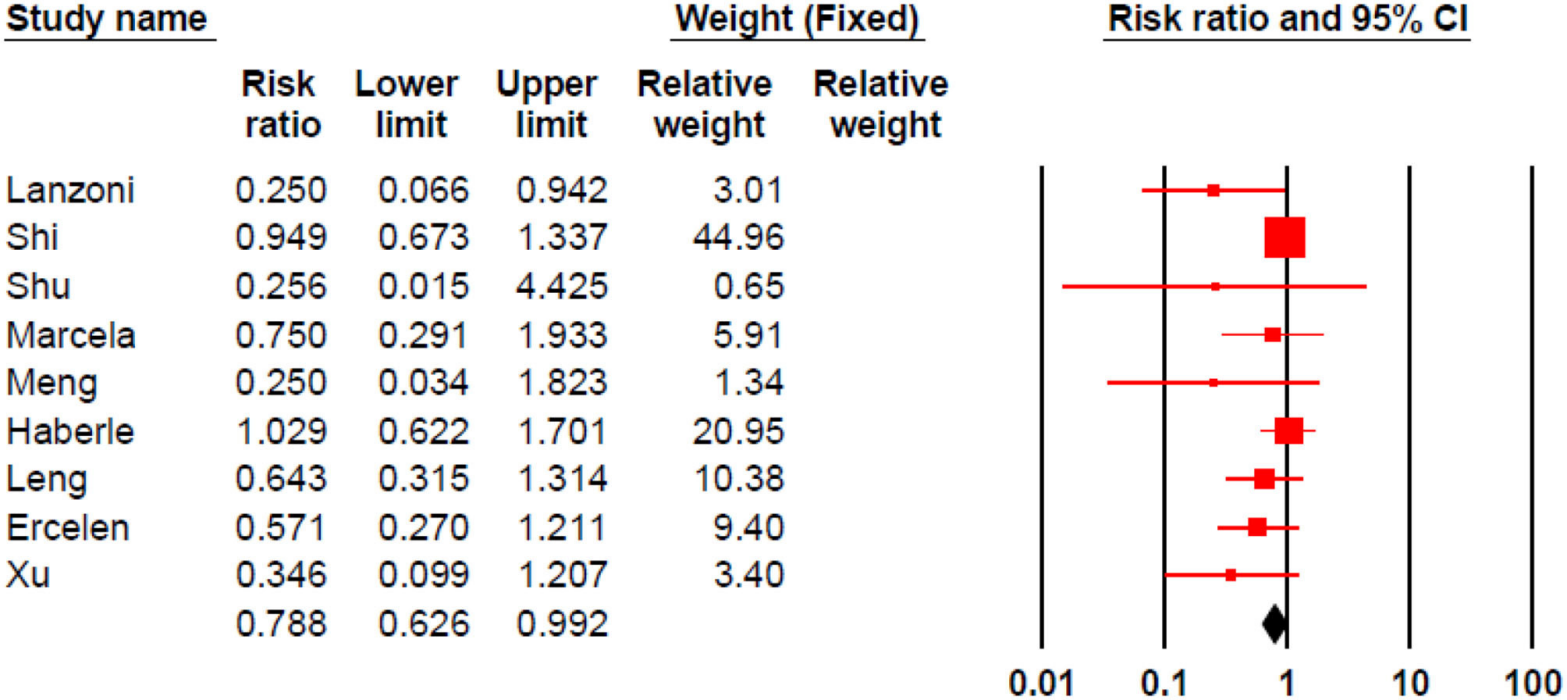
Pooled RR for morbidity analysis.

Stem cell therapy could significantly decrease morbidities in patients with COVID-19 (RR 0.788, 95% CI 0.626–0.992) (Figure 4).

**Figure 5:**
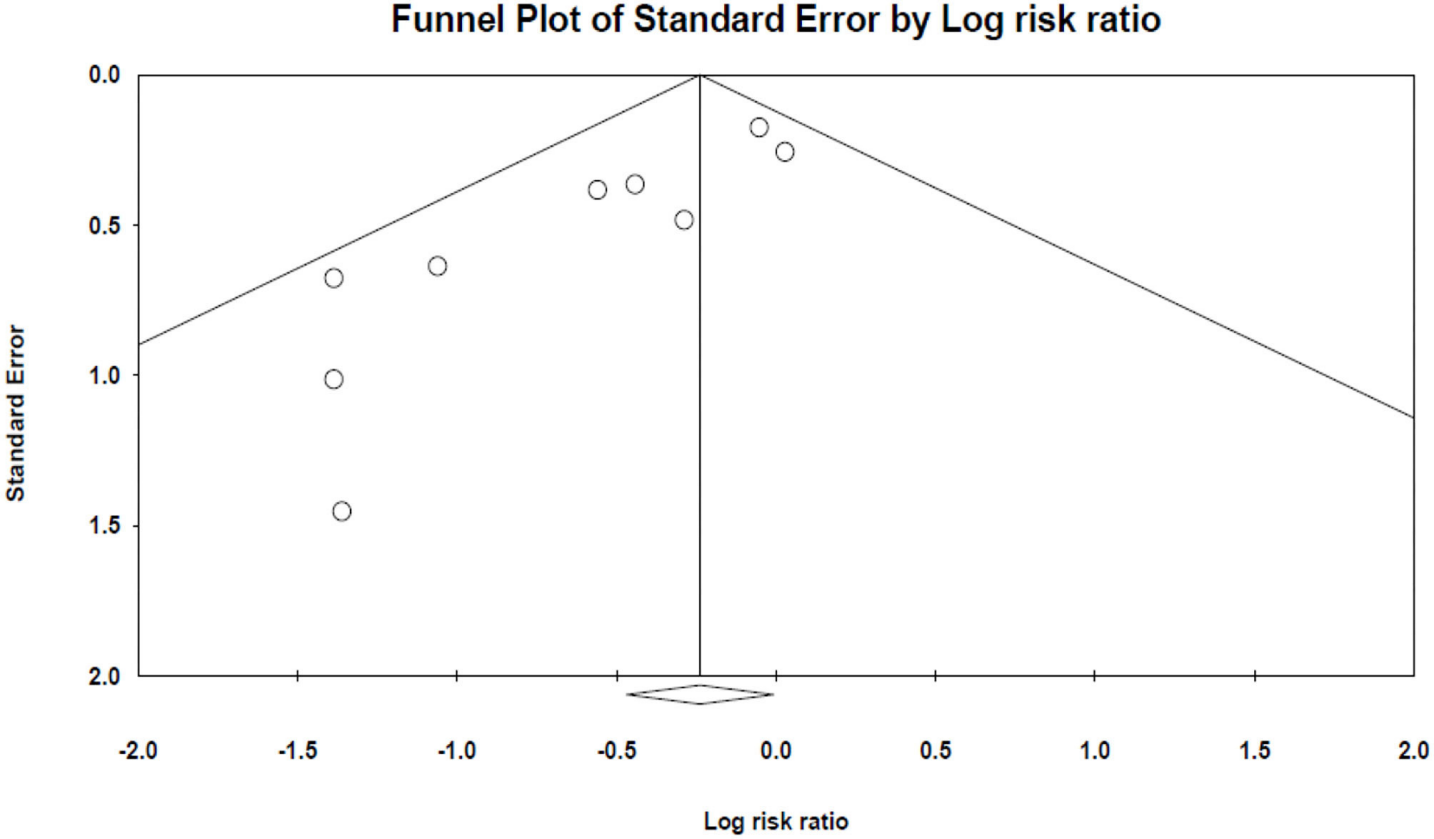
The funnel plot of morbidity analysis.

Egger's and Begg's tests indicated significant publication bias (The *P*-value of Egger's tests was 0.001, and Begg's tests was 0.047) (Figure 5).

### Pulmonary Function and Imaging Changes

Pulmonary function and imaging changes have been assessed in seven studies (18–22, 24, 25). These studies showed that stem cell therapy could improve O_2_ saturation, Murray score, and lung lesions (Table 6).

**Table 6:** Secondary outcome: efficacy.

**References**	**Mortality in stem cell (death/***n***)**	**Mortality in control (death/***n***)**	**Morbidity in stem cell (afflicted/***n***)**	**Morbidity in control (afflicted/***n***)**	**Pulmonary and imaging changes**	**Systemic changes and symptoms**	**Inflammatory markers**
Meng et al. (18)	0/9	0/9	1/9	4/9	CT images showed lung lesions entirely faded away within 2 weeks after MSC infusion, while lung lesions still existed in one severe patient in the control group at discharge	Clinical symptoms at discharge; respectively, for MSC and control group: (Fever: 5/9; 2/9), (Fatigue: 4/9; 5/9), (Cough: 4/9; 8/9) and (breath shortness: 1/9; 5/9). The period of admission to discharge was same in MSC and control group (20.00 vs. 23.00 days, *P* = 0.306)	There was a reduced trend in IFN-γ, TNF-α, MCP-1, IP-10, IL-22, IL-1RA, IL 18, IL-8, MIP-1 levels within 14 days in MSC group
Häberle et al. (19)	1/5	10/18	4/5	14/18	The MSC group had a higher Murray score on admission than control patients, reflecting more severe pulmonary compromise (3.5 + 0.2 vs. 2.8 + 0.3). At discharge, the MSC group showed a significantly lower Murray score than the control group (0.3 + 0.1 vs. 1.3 + 1.1)	ICU stay in control was less than the MSC group but not significant (*P* = 0.07) (due to the higher incidence of deceased patients within the control group and a higher percentage of ECMO use in the MSC group). The incidence of kidney injury and hepatic failure in the MSC group did not differ from the incidence in the control group of patients	The values for CRP and IL-6 did not differ significantly between the groups during ICU treatment. A significant reduction in leukocytes and neutrophils was found at discharge in the MSC group compared to the control group, showing a reduction of inflammation. A significant increase in lymphocytes at discharge was observed in the MSC group, suggesting that the acquired immune system is activated
Leng et al. (20)	0/7	1/3	4/7	3/3	2–4 days after MSC infusion, the O_2_ saturations rose to ≥95% at rest, without or with oxygen uptake (5 l/min)	2–4 days after MSC infusion, all the symptoms disappeared in all the patients	Reduction of pro-inflammatory TNF and increasing of anti-inflammatory IL-10 in serum was significant (*p* <0.05). The serum levels of IP-10 and growth factor VEGF were both increased but not significantly
Nesrin et al. (21)	4/8	2/3	4/8	3/3	In four patients, chest X-rays approved clinical improvement, and the need for O_2_ support was decreased, and they were discharged. Other four MSC patients remained in critical condition and died, although there was a significant improvement in their prognostic markers	The significant improvement in the efficacy outcome was not correlated with the clinical progress in four of eight MSC patients who passed away. Among the patients who survived until the end of the study (4 in case and 1 in control), all four patients in the case were discharged from ICU, and one patient in control still was in ICU	Compared to the baseline, there was a significant reduction in CRP (*p* = 0.036), Hb (*p* = 0.03), and fibrinogen (*p* = 0.012) values on post-treatment day 5. While there was an elevation in lymphocyte count between baseline and post-treatment, the change did not reach statistical significance (*p* = 0.06). There was no statistically significant change in ferritin, SaO_2_, RR, NC, troponin, and PC (*p* > 0.05) between baseline and post-treatment day 5
Xu et al. (22)	2/26	6/18	3/26	6/18	Dyspnea and SpO_2_ showed a significant improvement after MSC infusions. Chest imaging findings were improved in the MSC group in the first month after infusion	The average time taken to improve for the MSC group was 5.8 days shorter, significantly less than the control group (*P* = 0.049), showing that MSC infusion could shorten the time required for treatment. There was no significant	no significant differences observed in inflammatory markers including CRP (*P* = 0.486), IL-6 (*P* = 0.375) serum level before and after MSC transplantation
						difference in either the length of hospital stay, the number of days in ICU, the occurrence of shock or multiple organ failure between the two groups (*P* > 0.05 for all)	
Giacomo et al. (23)	2/12	7/12	2/12	8/12	NM	MSC infusion was associated with significantly improved patient survival (91 vs. 42%, *P* = 0.015), SAE-free survival (*P* = 0.008), and recovery time (*P* = 0.03)	In a comparison between groups at day 6, significant differences were observed in the concentration of IFN-γ, GM-CSF, IL-5, IL-6, IL-7, TNF-α, TNF-β, PDGF-BB, and RANTES (*P* <0.05); median values of these molecules were lower in the MSC group
Lei et al. (24)	0/65	0/35	37/65	21/35	In the evaluation of the solid component lesions, the total lung lesion proportion of the whole lung volume showed a significant decrease in the MSC group against the placebo group (*P* = 0.043)	6-min walking distance was longer in the MSC group than in the placebo group but not significant (*P* = 0.057). Other parameters including DLco and VCmax, the six-category scale, status of oxygen therapy, and mMRC dyspnea score were similar between the two groups	there was no significant difference in the subsets of peripheral lymphocyte counts (CD4^+^ T cells, CD8^+^ T cells, B cells, NK cells) and plasma biomarkers between the two groups
Shu et al. (25)	0/12	3/29	0/12	4/29	Chest CT scans approved that in the number of lobes involved, the CT scores, consolidation, and GGO in the MSC group were significantly better than those in the control group (*P* <0.05)	On day 14, 11 patients (91.67%) of the MSC group experienced obvious clinical symptom improvements, usually manifesting as obvious absorption on imaging and significant remission of dyspnea; however, only 15 patients (51.72%) of the control group felt symptom relief	CRP and IL-6 levels were significantly reduced from day 3 of MSC infusion, and the lymphocyte count gave back to normal levels in less time
Gina Marcela Torres et al. (26)	4/20	6/24	5/20	8/24	NM	The hospital stay period in the stem cell group was less than the control group (mean of 27.4 vs. 41.6 days). The interval from the intervention day until the discharge, the stem cell group had a maximum of 43 days compared with the control group with 125 days	In the stem cell group, the creatinine, WBC, neutrophil, and platelet count, did not show significant differences during the interval of study, but CRP and lymphocyte count were extremely low after the infusion
Dynasty (27)	0/20	0/10	0/20	0/10	NM	NM	NM

*CRP, C-reactive protein; CT, computed tomography; DLco, diffusion lung capacity for carbon monoxide; ECMO, extracorporeal membrane oxygenation; GGO, ground-glass opacity; GM-CSF, granulocyte-monocyte colony-stimulating factor; Hb, hemoglobin; ICU, intensive care unit; IFN, interferon; IL, interleukin; IL-1RA, interleukin 1 receptor type 1; IP-10, interferon-inducible protein 10; MCP-1, monocyte chemoattractant protein 1; MIP-1, macrophage inflammatory protein 1-alpha; mMRS, modified Medical Research Council Dyspnea Scale; MSC, mesenchymal stem cell; NK cell, natural killer cell; NM, not mentioned; PC, platelet count; PDGF, platelet-derived growth factor two B subunits; RANTES, regulated on activation, normal T expressed and secreted (CCL5); SAE, severe adverse event; SpO_2_, oxygen saturation; TNF, tumor necrosis factor; VC, vital capacity; VEGF, vascular endothelial growth factor; WBC, white blood cell*.

### Systematic Changes and Symptoms

Systematic changes were defined as any changes from the overall baseline status. Except for one study that had not reported these changes (27), these data are available for the other nine studies in Table 6.

### Inflammatory Cells and Cytokines

Except for one study that had not reported the changes in inflammatory cells and cytokines (27), these changes are available for the other nine studies in Table 6.

## Discussion

In the current study, we found that stem cells are safe and can significantly decrease the mortality and morbidity of patients with COVID-19. Stem cell infusion can also improve pulmonary function, ameliorate symptoms, and suppress inflammation.

Safety is the primary issue that should be considered for any therapy. In our included studies, none of the mortalities were related to cell infusion. Thompson et al. (28) showed that intravascular administration of MSCs is associated with an increased fever risk (RR = 2.48, 95% CI 1.27–4.86). One of the most common symptoms in patients with COVID-19 is fever. In our studies, just Meng et al. (18) reported that two patients with MSC developed fever immediately after infusion, which resolved without intervention within 4 h, and none of the other studies reported infusion-related fever. So it seems stem cell transplantation is safe in patients with COVID-19.

Our analysis showed that stem cell therapy could significantly reduce disease mortality and severity. Qu et al. (29) conducted a meta-analysis of human studies on acute respiratory distress syndrome (ARDS). ARDS is a significant cause of mortality in patients with COVID-19. They reported that MSC therapy could reduce mortality in patients with ARDS but was not statistically significant. This dissimilarity could be due to study design and baseline characteristics; we just included patients with COVID-19, but Qu et al. included patients with ARDS with various ARDS causes like COVID-19, influenza, sepsis, and aspiration.

Based on the evaluation of the pulmonary function, and inflammatory markers changes in the included studies, MSC therapy could reduce inflammation and enhance pulmonary function. Similarly, a systematic review by Mahendiratta et al. (30) suggests that MSCs are capable of reducing systemic inflammation and protecting patients against COVID-19 infection. It seems the mechanism of action is due to the immunomodulatory effect of MSCs; which involves: (a) inhibiting B cells, T cells, NK cells, and dendritic cells activation; (b) decreasing macrophage M1 and enhancing macrophage M2 activation; (c) inhibition of mast cell degranulation; (d) promoting T regulatory and Th2 cell (31).

Interleukin 6 (IL-6) is a prognostic marker in COVID-19 infection, and tocilizumab blocks its receptors, a drug of choice for COVID-19 infection (32). Our data show that MSCs can reduce IL-6 in serum, which means that MSCs can mimic tocilizumab by decreasing IL-6 activity. Das (33) proposes that the anti-inflammatory effect of MSCs is attributable to the ability to secreting lipoxin A4 (LXA4), PGE2, and their precursors, which prevent the production of pro-inflammatory cytokines like IL-6 and tumor necrosis factor-alpha (TNF-α); this feature could be engaged to confront “cytokine storm” that which is seen in COVID-19 infection.

One complication of COVID-19 is excessive lung fluid production and pulmonary edema, which disturb proper pulmonary function. MSCs release keratinocyte growth factor (KGF), angiopoietin-1, and LXA4 in their exosomes. These factors activate the Na+-K+ pump, thus reducing the permeability of the alveolar epithelium to proteins and fluid and inhibiting fluid accumulation in lung tissue and edema. Another complication of COVID-19 is lung fibrosis. This complication should be taken seriously because it is irreversible if it occurs. MSCs prevent lung fibrosis by two mechanisms: (a) differentiating to alveolar type II cells; (b) paracrine signals (like KGF) that induce proliferation and inhibit apoptosis in type II alveolar cells (11, 34).

An important question is: Whether MSCs can get infected by COVID-19? Researches have shown that COVID-19 uses angiotensin-converting enzyme 2 (ACE2) and transmembrane serine protease 2 (TMPRSS2) as receptors to enter cells and infect them. Avanzini et al. (35) and Schäfer et al. (36) in their *in vitro* studies found that both fetal and adult MSCs have a deficient expression of ACE2 and TMPRSS2. Hernandez et al. (37) reported the same result in human UC-MSCs. However, Desterke et al. (38) reported that adult MSCs express ACE2 highly, while placenta-derived MSCs express ACE2 at a low level and only in initial passages of cultures. Anyhow, it seems MSCs are resistant to get infected by COVID-19 by low expression of ACE2 and TMPRSS2, which will cause them to dodge the infection and perform their immunomodulatory functions.

The COVID-19 mortality rate is higher among immunocompromised patients (39), and as we discussed before, MSCs modulate the immune response. So MSCs infusion for an immunocompromised patient may exacerbate the infection. There is still no large-scale clinical trial that evaluates the safety and efficacy of cell infusion for COVID-19 infection in immunocompromised patients. However, many patients in our studies received corticosteroids during the trial, which suppressed the immune system, and cell infusion was still safe and effective. Cui et al. (40), in an *in vitro* study, found that coculturing of human MSCs and NK cells of immunocompromised patients can improve the function of impaired NK cells and enhance the synthesis of interferon-gamma (IFN-γ) (which is a crucial part of the innate immune response during viral infection). Also, Lim et al. (41) found that human MSCs reduce lung injury in severe combined immunodeficiency (SCID) mice but not in immunocompetent mice.

Unfortunately, there is no protocol for stem cell therapy in patients with COVID-19. So trials are heterogeneous in terms of stem cell source, culture, dose, delivery route, and even the stage of COVID-19 infection that MSCs are administered. Despite these heterogeneities in reviewed studies, they all reported stem cell therapy as a safe and effective treatment for patients with COVID-19. Large-scale RCTs with accurate methodology will help to develop the best protocol in this field.

Some limitations of this study should be taken into consideration. First, although we started with a large number of articles, after several screenings, the number of eligible studies was relatively small. This could have reduced the power of the conclusion. Second, the potential influence of preexisting conditions, the severity of infection, and the COVID-19 variants could not be investigated because of the limited information obtained from the studied articles. Third, as with any systematic review, limitations associated with potential publication bias should be considered. Furthermore, studies' variability, different patients' characteristics, different morbidity definitions, and wide range of outcome metrics for pulmonary changes and inflammatory indicators were other limitations.

## Conclusions

Our analysis demonstrated that stem cells are safe to use in patients with COVID-19 and could significantly reduce mortality and morbidity rate in infected patients. Due to the low number of included studies, a large-scale analysis is needed to measure the outcomes. Likewise, a protocol for stem cell therapy in COVID-19 infection should be defined to achieve the best possible clinical outcomes.

## Data Availability Statement

The original contributions presented in the study are included in the article/supplementary material, further inquiries can be directed to the corresponding authors.

## Author Contributions

The study was designed by MZ and MN. EA, SK, NT, and AA performed the search, study selection, and data extraction. Statistical analysis was performed by MN. The first draft of the manuscript was written by MZ, EA, SK, NT, and AA. MN and MZ revised the article. All authors contributed to the article and approved the submitted version.

## Conflict of Interest

The authors declare that the research was conducted in the absence of any commercial or financial relationships that could be construed as a potential conflict of interest. The reviewer AB declared a shared affiliation with the authors to the handling editor at the time of review.

## Publisher's Note

All claims expressed in this article are solely those of the authors and do not necessarily represent those of their affiliated organizations, or those of the publisher, the editors and the reviewers. Any product that may be evaluated in this article, or claim that may be made by its manufacturer, is not guaranteed or endorsed by the publisher.
